# Short RNA Guides Cleavage by Eukaryotic RNase III

**DOI:** 10.1371/journal.pone.0000472

**Published:** 2007-05-30

**Authors:** Bruno Lamontagne, Sherif Abou Elela

**Affiliations:** Groupe ARN (RNA Group), Laboratoire de Génomique Fonctionnelle (Laboratory for Functional Genomics), Département de Microbiologie et d'Infectiologie, Faculté de Médecine et des Sciences de la Santé, Université de Sherbrooke, Sherbrooke, Québec, Canada; Victor Chang Cardiac Research Institute, Australia

## Abstract

In eukaryotes, short RNAs guide a variety of enzymatic activities that range from RNA editing to translation repression. It is hypothesized that pre-existing proteins evolved to bind and use guide RNA during evolution. However, the capacity of modern proteins to adopt new RNA guides has never been demonstrated. Here we show that Rnt1p, the yeast orthologue of the bacterial dsRNA-specific RNase III, can bind short RNA transcripts and use them as guides for sequence-specific cleavage. Target cleavage occurred at a constant distance from the Rnt1p binding site, leaving the guide RNA intact for subsequent cleavage. Our results indicate that RNase III may trigger sequence-specific RNA degradation independent of the RNAi machinery, and they open the road for a new generation of precise RNA silencing tools that do not trigger a dsRNA-mediated immune response.

## Introduction

The capacity of short RNA duplexes to direct sequence-specific RNA degradation provides an almost universal tool for design-based gene silencing. This technique termed RNA interference (RNAi), is initiated by either endogenous RNA duplexes generated by members of the RNase III family (e.g. Drosha and Dicer)[1] or through the introduction of exogenous duplexes[2]. However, the components of the RNAi machinery, with the exception of RNase III[3], are not conserved in bacteria and certain eukaryotes including *Saccharomyces cerevisiae*. In these organisms, most documented gene specific mRNA degradation events including those performed by RNase III are not sequence but structure dependent[4]–[7].

Members of the RNase III family share a conserved dsRNA-binding domain (dsRBD) and a catalytic domain[3], [8]. In yeast there is only one isoform of RNase III (Rnt1p)[3] involved in the processing of several non-coding RNAs[9]–[11] and the degradation of a wide variety of mRNAs[5], [6]. Unlike most RNase IIIs, Rnt1p has reduced affinity for generic A-form helix and instead recognizes hairpins as short as 5 base pairs (bp) when capped with NGNN or AAGU tetraloops[12]. Rnt1p's substrates are cleaved 14 and 16 nucleotides (nts) away from the terminal tetraloop making Rnt1p a helical ruler [13], [14].

Since RNAi does not exist in *S. cerevisiae*, we asked whether there is an independent strategy to target specific RNA sequences for degradation by RNase III. Our hypothesis was that Rnt1p could function as an RNP complex and use a small RNA guide to cleave a specific RNA sequence. To test this hypothesis we generated RNA transcripts containing a 5 bp hairpin that binds Rnt1p fused to sequences complementary to different RNA targets. As predicted, the different RNA guides successfully bound to Rnt1p and directed a specific cleavage at a fixed distance from the RNA hairpin *in vitro* and reduced the expression of abundant nuclear RNAs *in vivo*. Together, our data indicate that RNase III may function as a sequence specific RNP complex and reveal a new approach for the regulation of nuclear RNA.

## Results

### Rnt1p does not require a complete RNA helix for cleavage

**Figure 1:**
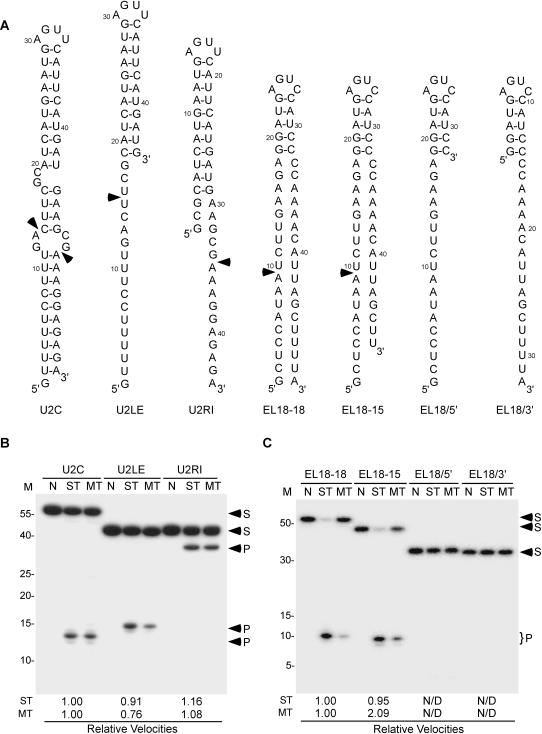
Rnt1p does not require a complete A-form helix for cleavage. (A) Schematic representations of Rnt1p substrates used in B and C. U2C, U2LE, and U2RI were derived from Rnt1p cleavage site at the 3′-end of U2 snRNA[9]. EL18-18, EL18-15, EL18/5′, and EL18/3′ are derived from the cleavage site at the 3′ end of U5 snRNA[18]. The arrowheads indicate major Rnt1p cleavage sites. (B) and (C) The different 5′-end labeled substrates were incubated in the absence (N) or presence of recombinant Rnt1p. Cleavage was carried out either in enzyme excess to measure the single turnover rate (ST) or in RNA excess to measure the multiple turnover rate (MT). The cleavage products were fractionated by 20% denaturing PAGE and visualized by autoradiogram. The cleavage efficiencies are presented as fractional velocities relative to the parental substrate. The values reflect the average of three independent experiments. The RNA marker (M) is indicated on the left. The positions of the cleavage products (P) and the substrates (S) are indicated by arrowheads on the right.

Most RNase IIIs and other dsRBPs identify their substrate by recognizing the distance between the minor grooves generated by one turn (i.e. 11 bp) of an A-form RNA helix[14]. In contrast, yeast Rnt1p has low affinity to duplex RNA and instead recognizes the fold of NGNN[15] or AAGU[16] tetraloops suggesting that this enzyme may not require the conventional 11 bp duplex for cleavage[12], [14]. In order to determine the minimum length of the RNA duplex required for Rnt1p cleavage, we generated a series of RNA transcripts with a fixed tetraloop sequence but with different single and double-stranded RNA lengths (Figure 1A). The sequence of the first three substrates was based on the Rnt1p cleavage signals found at the 3′-end of U2 snRNA because it was previously shown that this RNA could be cleaved even when the cleavage site is unpaired[9]. The cleavage efficiencies of the different substrates were compared under single (trace RNA amount) and multiple (1:8 protein excess) turnover conditions (Figure 1B). As expected, the U2 3′-end model substrate (U2C) was cleaved by Rnt1p in all conditions at the expected fixed distance 14 and 16 nucleotides from the terminal tetraloop (Figure 1B). Deletion of the nucleotides in the stem at the 3′-end of the tetraloop (U2LE), which leaves an eleven base-pair stem linked to an 18 nucleotide-5′ extension, was cleaved by Rnt1p once at 14 nts from the terminal tetraloop (Figure 1B). Similarly, the deletion of the 5′-end (U2RI) did not inhibit the cleavage at the 3′-end extension. The cleavage kinetics of these different substrates indicate that reducing the duplex length reduces Rnt1p's turnover but increases catalytic efficiency (Table 1). These data confirm that Rnt1p can cleave single-stranded RNA and suggests that long RNA duplexes are not required for cleavage.

**Table 1:** Kinetic parameters of Rnt1p cleavage of U2 snRNA 3′-end stem-loop derivatives

Substrate	k_cat_ (min^−1^)	K'_M_ (µM)	k_cat_/K'_M_ (L•min^−1^•µM^−1^)
U2C	0.348	1.563	0.223
U2LE	0.112	0.305	0.368
U2RI	0.199	0.449	0.444

The K'_M_ and k_cat_ values were determined by measuring the initial rate of production of the cleavage product as a function of substrate concentration. The calculations were performed using Michaelis-Menten equations. The indicated values represent the average of three independent measurements using 5′-end labeled substrates. The maximum k_cat_ error limits are±0.04 min^−1^, the K'_M_ error limits are±0.1 µM and the k_cat_/K'_M_ error limits are±0.05 L•min^−1^•µM^−1^.

RNA footprinting, chemical interference, and binding assays indicate that a minimum of a 5 bp stem capped with a NGNN tetraloop is required for Rnt1p binding[16], [17]. However, it is not clear whether the binding of this 5 bp short stem reflects the natural mechanism of substrate selection or arises from a non-specific or unproductive mode of binding. To differentiate between these two possibilities, we synthesized RNA substrates derived from a cleavage site with a known tertiary structure[15], [18]. The engineered substrates consist of U5 snRNA 5 bp stem[18] attached to heterologous ssRNA extensions at their 5′- (EL18/5′), 3′- (EL18/3′) or at both ends (EL18-18 and EL18-15) (Figure 1A). Interestingly, Rnt1p only cleaved substrates with two single-stranded RNA extensions (EL18-18 and EL18-15). (Figure 1C). Additional assays using a variety of substrates indicated that a minimal 9 and 11 nucleotide extensions at the 5′- and 3′-ends respectively are required for cleavage (data not shown). This indicates that RNA helices longer than 11 bp are not required for cleavage by Rnt1p.

### Directing Rnt1p cleavage using an RNA guide

**Figure 2:**
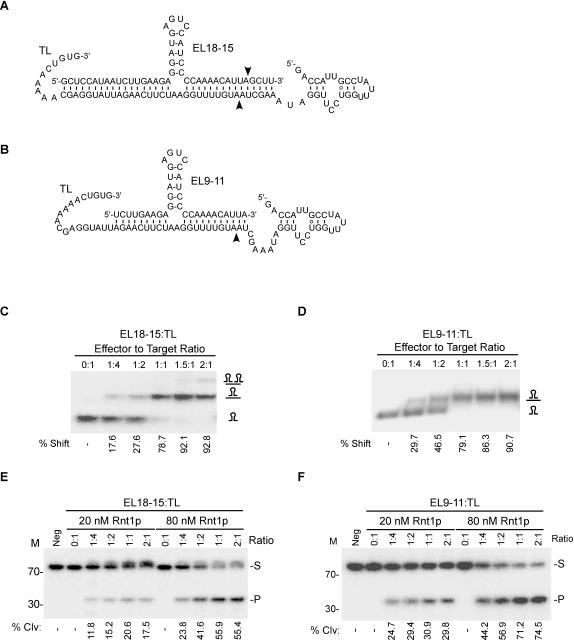
Rnt1p cleaves intermolecular RNA substrates. Illustrations of an RNA guide that could be cleaved by Rnt1p (EL18-15) (A) or a guide that cannot be cleaved by Rnt1p (EL9-11) (B) when in complex with the target RNA (TL). The arrowheads indicate the positions of the observed cleavage sites within the guide and target sequences. (C) and (D) illustrate the gel shift assay used to monitor the interaction of EL18-15 and EL9-11 with the target sequence (TL). The reaction products were loaded on a 12% non-denaturing PAGE. The complex formation was quantified using the Instant Imager and the average percent shift (%) obtained from two independent experiments is indicated below each gel. The complexes are indicated on the right. RNA cleavage was assayed using EL18-15 (E) or EL9-11 (F). The RNA was incubated with different ratios of 5′-end labeled target (TL) and two different Rnt1p concentrations (20 and 80 nM) for 20 minutes. The cleavage products were analyzed by 20% denaturing PAGE and the bands were quantified using Instant Imager. Average percent (%) cleavage of three independent experiments is indicated below each gel. The RNA marker is displayed on the left. The positions of the substrate (S) and product (P) are showed on the right.

The capacity of Rnt1p to form a stable complex with short RNA hairpins underscores the capacity of this enzyme to form RNP complexes under certain conditions. Rnt1p/RNA complexes are catalytically active since they mediate cleavage when attached to single-stranded RNA extensions (Figure 1). This is reminiscent of known RNP complexes like the snoRNP[19] and the RISC complexes[20] that use RNA as a guide to modify or cleave an independent RNA molecule *in trans*. Therefore, Rnt1p cleavage may also be guided by short RNA transcripts. To test this possibility, we generated RNA transcripts that contain an Rnt1p binding signal (5 bp stem) fused to RNA extensions complementary to the sequence of an independently transcribed single-stranded RNA fragment. One guide had long extensions (EL18-15) to allow cleavage in both guide and target RNA (Figure 2A) while the other had short extensions (EL9-11) to allow cleavage only in the target RNA (TL) (Figure 2B). As expected, a gel shift assay indicated that both guides bound their 72 nts long RNA substrate (TL) with similar efficiency (Figures 2C and D). The short guide EL9-11 readily formed a complex with its targeted RNA, while the long guide EL18-15 formed complexes only when the RNA mixture was pre-heated. This suggests that 20 nt long complementarities between the guide (EL9-11) and its target RNA are sufficient for complex formation. On the other hand, additional extensions in the guide sequence may increase the chance of intramolecular secondary structure, which may occlude target identification.

The ability of both guides to direct Rnt1p cleavage was tested by incubating them with 5′-end labeled target RNA (TL) and recombinant Rnt1p (Figures 2E and F). As shown, Rnt1p cleaved the substrate 14 nt from the guide tetraloop releasing a 33 nt product. However, no cleavage in the substrate was detected at the second predicted cleavage site 16 nt from the guide tetraloop (54 nt product). The inability of Rnt1p to cleave the second predicted cleavage site is not surprising since Rnt1p is known to bind to its substrate asymmetrically and is only tolerant of structural variations in one side of the tetraloop [12], [16], [17]. It is interesting to note that the uncleavable RNA guide (EL9-11) was more efficient in directing cleavage than the long cleavable RNA guide since uncleavable guide may be recycled to induce more than one round of target cleavage. We conclude that Rnt1p may use an RNA guide to cleave an independent RNA target.

### The guide RNA supports standard cleavage kinetics

**Figure 3:**
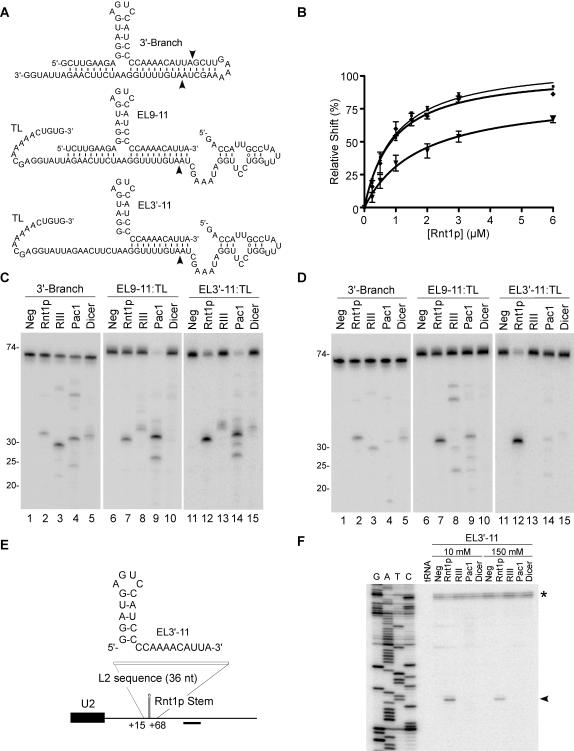
Comparison between inter- and intra-molecular RNA cleavage by different RNase IIIs. (A) Illustration of the different substrates used in C and D. 3′-Branch indicates a substrate allowing intramolecular cleavage by Rnt1p. EL3′-11 and EL9-11 indicate respectively a guide RNA with a single or two target complementary extensions. The target is indicated by TL. The arrowheads indicate the position of the observed cleavage by Rnt1p. (B) Quantitative analysis of RNA binding to Rnt1p. Increasing concentrations of Rnt1p (0.25 to 6 µM) were incubated with 3 fmol of 3′-Branch (▪), EL9-11:TL (σ) and EL3′-11:TL (τ) and the binding percentage (%) was plotted against the protein concentration. The curve fits were obtained using the Graph Pad Prism 4.0 program. Each data point is an average of four experiments. The target RNA in the *trans* reactions and the *cis* RNA were 5′-end labeled and incubated with members of the RNase III family. Rnt1p, bacterial RNase III (RIII), Pac1 and human Dicer were incubated in RNA excess under a 10 mM (C) or 150 mM (D) KCl. The position of the RNA ladder is shown on the left. (E) Sketch of a 36 nt fragment containing sequences complementary to EL3′-11 inserted into a U2 3′-end flanking region to replace a canonical Rnt1p substrate. The position of the oligonucleotide used for primer extension is indicated. (F) Mapping the cleavage of the U2 3′-end region with RNase IIIs. Yeast total RNA (20µg) from YHM111-U2L2 was incubated with EL3′-11 and RNase IIIs in 10 and 150 mM KCl. A primer complementary to the 3′-flanking sequence of U2 snRNA was extended in all cleavage reactions. The reference DNA sequence is shown on the left. The arrowhead indicates a specific cleavage product. The asterisk indicates a secondary structure at the mature U2 3′-end.

The biological significance of the guide/Rnt1p complex and its potential as an effective tool for gene silencing depends on the cleavage efficiency of this complex. To evaluate the efficiency of the guide driven cleavage, we compared it to that generated using standard Rnt1p substrates[14]. Binding and cleavage parameters were monitored using the EL9-11 guide, a shorter version that pairs with the target using only one 3′-end extension (EL3′-11), or a long 3′-Branch based substrate allowing classical Rnt1p cleavage *in cis* (Figure 3A). The RNA/protein complexes were resolved using a standard gel mobility shift assay, and complex formation was quantified and plotted as a factor of protein concentration (Figure 3B). The classical substrate (3′-Branch) and the guide RNA with two extensions (EL9-11) bound to Rnt1p with a similar apparent dissociation constant (K'_d_) of about 0.80 µM, while the guide RNA with only one single target complementary extension bound less efficiently with a K'_d_ of 1.9 µM (Table 1). This data suggest that decreasing the single-stranded RNA extension length decreases the affinity to Rnt1p perhaps by inhibiting interactions fostered by deleted sequences.

The cleavage efficiencies of the different substrates were tested by incubating each of them with Rnt1p and Mg^2+^. The target RNA was labeled at the 5′-end to track the product generation under low (Figure 3C) and physiological salt concentrations (Figure 3D). Rnt1p cleaved all three substrates at the predicted site 14 nts from the tetraloop with efficiency close to that of previously tested natural cleavage sites (regardless of the salt conditions)[3], [14], [17]. The guide's ability to direct Rnt1p cleavage was dependent on the presence of the conserved NGNN tetraloop (data not shown). The cleavage kinetics induced by the EL3′-11 were compared to that generated *in cis* within the 3′-Branch RNA (Table 2). Surprisingly, the guide-based cleavage exhibited about a 3-times higher K'_M_ and a faster k_cat_ than *cis* cleavage, while its specificity constant (k_cat_/K'_M_) was slightly reduced. We conclude that guide-mediated and classical Rnt1p substrates observe similar kinetic parameters.

**Table 2:** Kinetic parameters of Rnt1p cleavage of inter- and intra-molecular substrates

Substrate	K'_d_ (µM)	k_cat_ (min^−1^)	K'_M_ (µM)	k_cat_/K'_M_ (L•min^−1^•µM^−1^)
3′-Branch	0.75	0.303	0.125	2.423
EL3′-11:TL	1.90	0.629	0.406	1.548

The K'_M_ and k_cat_ values were determined by measuring the initial rate of production of the 34 and 33 nt cleavage products of 3′-Branch and EL3′-11:TL respectively, as a function of substrate (or complex) concentration. The calculations were performed using the equation one site binding (hyperbola) from Prism 4.0 (GraphPad) and the Michaelis-Menten equations. Errors in the values of the K'_d_ are within±0.10 µM. The indicated values represent the average of three independent measurements using 5′-end labeled substrates. The maximum k_cat_ error limits are±0.07 min^−1^, the K_M_ error limits are±0.05 µM and the k_cat_/K'_M_ error limits are±0.09 L•min^−1^•µM^−1^.

Guide-induced RNA cleavage may be a unique feature of Rnt1p or it may be shared by other members of the RNase III family. We examined the guide's capacity to induce target cleavage by RNase IIIs from bacteria (RNase III), fission yeast (Pac 1) and human (Dicer) (Figures 3C and D). As expected, all enzymes cleaved their natural substrates suggesting that all enzymes are active (data not shown). Interestingly, the different enzymes were capable of cleaving the 3′-Branch at both salt concentrations (Figure 3C and D, lanes 2–5) albeit with different efficiencies. This suggests that most RNase IIIs can tolerate a three-way junction and may cleave substrates with less than 16 consecutive base pairs. However, only Rnt1p was able to cleave the 3′-Branch substrate at a fixed distance from the NGNN tetraloop (Figures 3C and D, lane 2 and data not shown). The guide RNA that forms 19 bp with the target sequence (EL9-11) induced cleavage by Rnt1p, bacterial RNase III, and Pac1 in both low and high salt concentrations (Figures 3C and D, lanes 7–10). RNase III and Pac1 cleavages were not nucleotide or loop specific and the cleavage sites were different in different salt conditions (Figure S1). Surprisingly, at low salt concentration all enzymes cleaved the target RNA in the presence of EL3′-11 that forms only 11 base pairs with the target (Figure 3C, lanes 12–15). At high salt concentration, very little EL3′-11 dependent cleavage was detected except when Rnt1p was present (Figure 3D, lanes 12–15). These data indicate that with the exception of Rnt1p, most RNase IIIs require a duplex longer than one turn of a helix to support intermolecular RNA cleavage.

To evaluate the potential of guide RNA *in vivo*, we tested its capacity to identify a given sequence in a natural mixture of yeast total RNA. The Rnt1p cleavage signal at the 3′-end of pre-U2 snRNA was replaced by a sequence complementary to the EL3′-11 guide and the new U2/target construct was expressed *in vivo* (Figure 3E). In this way the processing of the U2 3′-end that is normally carried out by Rnt1p *in cis* [9] can only take place if the guide induces Rnt1p cleavage *in trans*. Total RNA was extracted from yeast expressing the U2/target transcript and incubated *in vitro* with the guide EL3′-11 and Rnt1p, bacterial RNase III, Pac1, or Dicer. The cleavage site in each case was visualized either by northern blot (data not shown) or by primer extension (Figure 3F). The U2/target RNA was cleaved by Rnt1p producing a single nucleotide cleavage 14 nucleotides from the guide tetraloop as predicted. The failure of Pac1, bacterial RNase III, and Dicer to cleave the U2/target could be explained by problems in target accessibility or competition with other imperfect duplexes in the yeast transcriptome. We conclude that guide RNAs may specifically select Rnt1p targets in a complex mix of natural RNA.

### The guide RNA restores cleavage of mutated Rnt1p substrate *in vivo*

**Figure 4:**
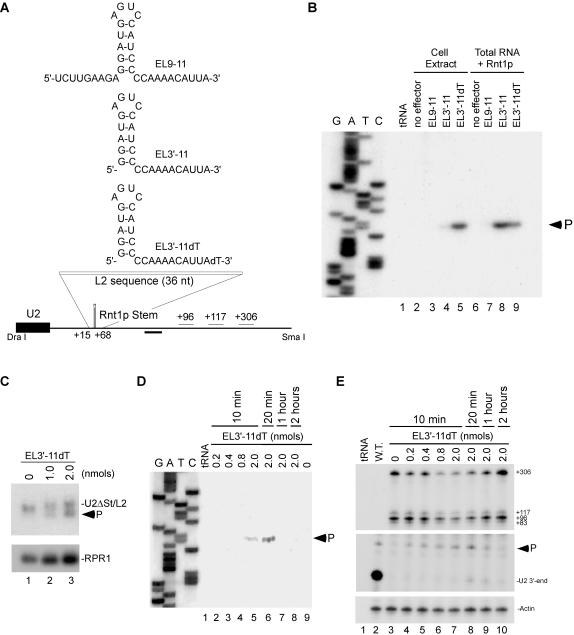
Guide RNA restored cleavage to a mutated Rnt1p cleavage site *in vivo.* (A) Secondary structure of RNA guides complementary to a mutated Rnt1p cleavage site at the 3′-end of U2 snRNA (L2). The position of the oligonucleotide used for primer extension is indicated below as well as putative poly(A) signals (+96, +117, and +306). (B) RNA guides were incubated in yeast extract or with yeast total RNA and recombinant Rnt1p for 20 min. The cleavage site was mapped using primer complementary to the 3′-flanking sequence of U2 snRNA. The reference DNA sequence produced using the same primer is shown on the left. The product corresponding to the cleaved RNA is indicated. Bacterial tRNA was used as negative control for the primer extension. (C) Yeast strain YHM111-U2L2 was electroporated with EL3′-11dT and the RNA extracted after 10 minutes of incubation. The RNA bands were analyzed by northern blot using a probe complementary to mature U2 snRNA sequence. A probe directed against RPR1 was used as loading control. The arrowhead indicates the position of the cleavage product. (D) Cleavage site mapping of yeast YHM111-U2L2 electroporated with EL3′-11dT. Total RNA was extracted between 10 minutes and 2 hours post-electroporation and annealed to the primer used in B. The reference DNA sequence is shown on the left. The product corresponding to the cleaved RNA is indicated. (E) Analysis of U2 snRNA 3′-end formation. RNA samples described in D were hybridized to an RNA probe (DraI-SmaI fragment) complementary to the 3′- flanking sequences of U2 snRNA, and digested with RNase T1. The mature U2 3′-end and the ends of the extended forms are indicated on the right. The Rnt1p-directed cleavage product is indicated by an arrowhead. The position of the different 3′-ends detected is indicated using wild-type U2 sequence as reference. A probe against actin was used as internal control for loading and quantification.

Guide RNA's potential to regulate gene expression was evaluated by examining their capacity to restore cleavage to mutated Rnt1p cleavage site in both cell extract and in total RNA (Figure 4). The guide EL9-11 with 2 single stranded extensions did not cleave the U2/target in cell extract (Figure 4B, lane 3) and only induced very weak cleavage in total RNA (Figure 4B, lane 7). In contrast, EL3′-11 with 1 single-stranded extension induced strong cleavage in total RNA extracted from yeast (lane 8) but not in cell extract (lane 4). We reasoned that the weak activity in cell extracts was due to the guide instability. Indeed, labeled EL3′-11 is readily degraded in cell extract (data not shown). To enhance the guide RNA stability in cell extract and later *in vivo*, we generated EL3′-11 RNA with an inverted deoxythymidine (dT) at the 3′-end. The addition of an inverted deoxythymidine reduces 3′ to 5′ exonuclease attack[21]. The modified guide (EL3′-11dT) directed efficient cleavage in cell extract (lane 5) and was stable in cell extract for up to 2 hours while unprotected EL3′-11 degraded after 10 minutes (data not shown). Moreover, Northern blot analysis indicated that as little as 1 nmol of EL3′-11dT could induce the cleavage of up to 50% of the U2/target in 20 minutes (Figure 4C).

In order to assay the guide activity *in vivo*, we had to establish a method for RNA transfection. To do this, we adapted an electroporation based transformation strategy that is normally used for DNA transformation[22]. Different concentrations of EL3′-11dT were transfected into yeast cells and total RNA was extracted after different incubation times. The guide-dependent cleavage product was monitored by primer extension complementary to the sequence downstream of the predicted cleavage site. As shown in figure 4d, a single 5′-end corresponding to predicted cleavage product was detected in RNA extracted from cells transfected with 2 nmol EL3′-11dT after 10 minutes (lane 5) but not in the control cell transfected with water (lane 9). The cleavage product increased after 20 minutes of incubation (lane 6) but disappeared after one hour (lane 7) as expected since Rnt1p cleavage products are highly unstable[9].

Rapid RNA processing and difficulties detecting unprocessed RNA precursors suggest that RNA processing takes place co-transcriptionally. However, the importance of co-transcriptional RNA cleavage to RNA maturation remains unclear. We have taken advantage of the newly developed guide technology to assess whether the co-transcriptional Rnt1p cleavage of the pre-U2 3′-end is required for U2 maturation. We monitored the generation of mature U2 snRNA from a transcript that depends on the guide EL3′-11dT for cleavage (Figure 4E). As expected, mainly mature U2 was detected in wild type cells (lane 2). In contrast, cells expressing U2/target accumulated unprocessed U2 and no mature 3′-end was detected (lane 3). Electroporation of different concentrations of guide induced cleavage in the target sequence decreasing the amount of the U2/target precursors (top panel). A product cleaved at the primary cleavage site of Rnt1p[9] was detected in a guide-dependent manner (e.g. lanes 4–8). However, only a small amount of mature 3′-end was observed after the guide electroporation even after 2 hours (lanes 8–10). Since the reduction in RNA precursors does not lead to corresponding accumulation of mature U2, we conclude that most of the guide-dependent cleavage events do not generate stable RNA. This data clearly demonstrate that the processing efficiency of non-coding RNA depends on the nature and timing of the endonucleolytic cleavage initiating the maturation process.

### Guide-specific cleavage of natural RNAs

**Figure 5:**
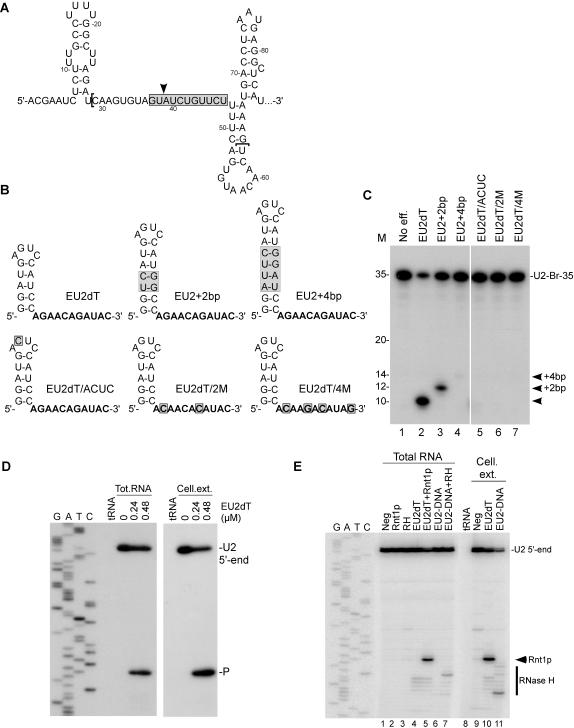
Guide RNA directs sequence specific cleavage in a natural RNA sequence. (A) Secondary structure of the U2 snRNA branch site region (nucleotides 1 to 86). The gray box, the arrowhead and the brackets represent respectively the targeted region by Rnt1p, the anticipated cleavage site by Rnt1p and the region used for *in vitro* cleavage assays (U2-Br-35). (B) Sketches representing the secondary structure of guides recognized by Rnt1p and complementarity to the U2 branch site. Sequences in bold represent the nucleotides complementary to the U2 target. The gray boxes indicate mutations relative to the control (EU2dT). (C) *In vitro* cleavage of 5′-end labeled U2-Br-35 with Rnt1p and the different RNA guides. The cleavage reactions were performed in RNA excess with a guide/target ratio of 1:1. The positions of the cleavage products are indicated on the right and the RNA marker is displayed on the left. (D) Total yeast RNA and recombinant Rnt1p or yeast cell extract prepared from strain YHM111-U2L2 were used to analyze Rnt1p-directed cleavage using EU2dT. Primer complementary to the 3′-flanking sequence of the U2 snRNA branch site was extended on the extracted RNA to map the cleavage site. The reference DNA sequence produced using the same primer is shown on the left. The product corresponding to the cleaved RNA and the U2 5′-end are indicated on the right. Bacterial tRNA was used as negative control for the primer extension. (E) Cleavage comparison between Rnt1p and RNase H in total RNA or cell extract prepared from yeast YHM111-U2L2. The cleavage specificity was determined by primer extension. The reference DNA sequence produced using the same primer is shown on the left. The RNA guide and the DNA oligo used with RNase H targeted the same nucleotides. The positions of Rnt1p and RNase H cleavage products and the U2 5′-end are indicated on the right.

To evaluate the guide cleavage strategy as a tool for gene silencing, we designed and tested a series of guide RNAs targeting the branch site of U2 snRNA[23] (Figures 5A and B). Guides with different stem lengths and loop structures were used to demonstrate cleavage specificity (EU2dT, EU2+2bp and EU2+4bp). As expected, a guide RNA with a 5 bp stem and 11 nt complementary to the targeted U2 branch site (EU2dT) induced a substantial cleavage when incubated with total RNA and recombinant Rnt1p (Figure 5C, lane 2). Increasing EU2dT stem length by two base pairs (EU2+2 bp) shifted cleavage by 2 nt (lane 3). Insertion of an additional 2 bp (EU2+4 bp) shifted cleavage further by two nucleotides, while strongly reducing cleavage efficiency (lane 4). The guide EU2+4 bp is long enough to be directly cleaved by Rnt1p *in cis* independent of the target, which explains the reduced efficiency of target cleavage. These results indicate that RNA guides may act as helical scales marking the distance to the cleavage site. As expected, guide RNA with a mutation in the conserved second base of the Rnt1p tetraloop (EU2dT/ACUC) blocked cleavage (lane 5). Mutations altering the guide homing sequence complementary to the target site (EU2dT/2M and EU2dT/4M) also blocked cleavage (lanes 6 and 7) confirming the cleavage specificity.

EU2dT was chosen for subsequent assays in cell extract and *in vivo* based on its performance in the *in vitro* cleavage assay. Incubation of EU2dT with total RNA and recombinant Rnt1p or cell extract introduces a single cleavage site at the predicted distance from the loop within the U2 branch site (Figure 5D) indicating that this guide is both specific and stable even in the presence of total yeast RNA, proteins and ribonucleases. To accurately evaluate the value and efficiency of the guide RNA-mediated cleavage, we wanted to compare it to another enzymatic activity that targets sequence specific endonucleolytic cleavage *in trans*. RNase H DNA-mediated cleavage of RNA has been exploited as a tool for gene silencing[24] and is routinely used to cleave RNA *in vitro* [25]. Therefore, we chose RNase H as a benchmark for evaluating the utility of Rnt1p. We directly compared the performance of Rnt1p/EU2dT to an RNase H/DNA oligonucleotide (EU2-DNA) targeting the same sequence in total RNA and cell extract (Figure 5E). Both oligonucleotides induced cleavage of the targeted U2 snRNA when present in total RNA (lanes 5 and 7). However, unlike with Rnt1p, the scissile bond of RNase H cleavage was difficult to predict. In cell extract, EU2dT induced the cleavage of a single phosphodiester bond (lane 10), while the EU2-DNA induced a heterogeneous cleavage pattern (lane 11). The comparatively higher precision of Rnt1p was confirmed by other experiments using guides and DNA oligonucleotides targeting a variety of RNA transcripts (data not shown). At high salt concentrations the Rnt1p guide may support structure sensitive cleavage like RNase H where only single-stranded RNA is cleaved, and thus it may be used as a probe for RNA structure (data not shown). However, unlike RNase H, Rnt1p guide may also act as an RNA restriction enzyme allowing structure independent cleavage at low salt concentration (data not shown) increasing its utility as gene silencer. We conclude that Rnt1p provides an effective alternative to RNase H as a probe for RNA structure and as an RNA restriction enzyme.

### Inhibition of gene expression using guide RNAs

**Figure 6:**
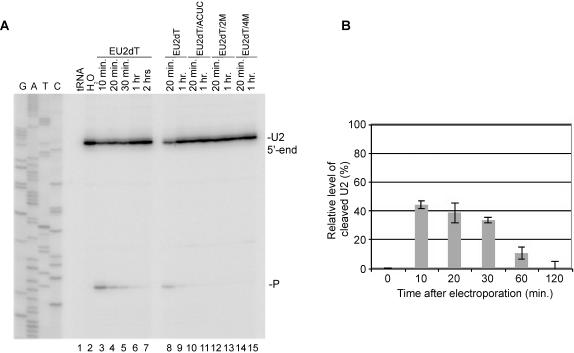
Small RNA guides induce Rnt1p cleavage *in vivo.* (A) Mapping the RNA-directed cleavage of U2 from living yeast cells. Yeast cells were electroporated with 2 nmols of EU2dT or mutant RNA guides and total RNA was extracted between 10 minutes and 2 hours after electroporation. The primer PE-U2-Br was annealed with the RNA samples and extended on U2 snRNA. The corresponding DNA sequence is shown on the left. (B) Relative levels of cleaved U2 snRNA after electroporation were established using RNA extracted in A and loaded on 1.2% agarose-formaldehyde gel, transferred to a nylon membrane and hybridized with probes specific for nucleotides 7 to 29 into mature U2 or against the 25S rRNA to serve as internal control and visualized by Phosphor Imager. The intensity of each band was quantified using Image Quant 5.0. The levels of U2 snRNA were normalized against the 25S rRNA and the electroporation efficiency (53±8%), and were plotted as a function of time after electroporation with EU2dT.

To examine the potential of Rnt1p guides as regulators of gene expression *in vivo*, we electroporated EU2dT guide into yeast cells and monitored both the transfection efficiency and the degradation of U2 snRNA. After electroporation, equal cellular distribution of 5′-fluorescein labeled EU2dT (EU2dT-Fl) was observed in 53±8% of the cells (data not shown). Therefore, the maximum expected inhibition level of the targeted RNA by any electroporated guide is about 50%. Induction of U2 snRNA cleavage by EU2dT *in vivo* was monitored by primer extension. As shown in figure 6a, electroporation of EU2dT generated cleavage product after a 10 minute incubation. Increasing the incubation period resulted in the degradation of the cleavage product (lanes 3 to 7). Consistent with the estimated half-life of the guide RNA, the amount of intact U2 in treated cells was restored to pre-treatment levels after 2 hours of incubation. Mutations altering the Rnt1p binding site (EU2dT/ACUC) or the sequence complementary to U2 (EU2dT/2M and EU2dT/4M) blocked cleavage (lanes 10–15) confirming the reaction specificity. Northern blot quantification demonstrated that more than 40% of U2 RNA in electroporated cells was degraded after 10 minutes (Figure 6B). Increasing the incubation time beyond 30 minutes gradually restored U2 expression presumably due to the degradation of the guide. Moreover, transfection of mutated guide RNA left the level of U2 snRNA unchanged (data not shown). The capacity to degrade nearly half of the highly expressed U2 snRNA (200–500 molecules per haploid cell[26]) in 10 minutes is a clear indication of the efficiency of the guide as a tool for gene regulation. We conclude that Rnt1p guides are efficient tools for nuclear site-directed RNA degradation *in vivo*.

## Discussion

RNAi and RNA turnover are often treated as two distinct mechanisms of gene regulation despite the fact that RNAi is in essence a process of targeted RNA degradation. The sequence specificity of RNAi-based gene silencing and its induction by small RNA *in trans* makes it appear quite different from the largely non-specific ribonuclease-based RNA degradation. In this study, we have shown that a classical endoribonuclease may use small RNA guides for sequence specific cleavage in analogy to the RNAi degradation mechanism. Rnt1p, a member of the RNase III family, was shown to form an RNP complex with a specific RNA hairpin and uses it to guide the cleavage of targeted RNA *in trans*. This feature allowed us to transform Rnt1p from a structure-based enzyme into a sequence-specific RNP, supporting evolutionary models explaining the origin of RNA guided protein complexes. The capacity of short and largely single-stranded RNA to guide RNase III cleavage demonstrates the flexibility and tremendous potential of these enzymes as regulators of gene expression.

### Evolution of an RNA-protein complex

The debate about the evolutionary origin of RNA-protein complexes started soon after the discovery of the first catalytic RNA[27]. The protein dominance of modern enzymatic activity led to the belief that RNAs are ancient relics of catalysis[28]. It was proposed that ancient RNA enzymes were taken over by gradually evolving proteins creating in the process several intermediates incorporating both RNA and proteins moieties[29], [30]. In this “RNA first” model of RNP, an RNA molecule with an established function would recruit a protein to enhance activity. However, the recent explosion in the discovery of small RNAs that guide protein functions ranging from rRNA modification to translation repression[31] started to paint a different story. It is now argued that many guide RNAs including C/D and H/ACA box snoRNAs evolved from pre-existing RNA that acquired affinity for ancient proteins and used it to target their function[32]. The “protein first” model of RNP argues that proteins with established functions scavenged non-functional or duplicated RNA transcripts for better or modified substrate specificity. The protein first model could be easily extended to components of the RNAi machinery[1] that includes classical ribonucleases like RNase III[3] and small RNAs. Many small RNAs discarded from introns could guide sequence specific cleavage *in trans* [33]. The ability of small RNA to guide cleavage by Rnt1p, the yeast orthologue of RNase III (Figure 2), directly supports the basic notion of the protein first model of RNP. Indeed, the work presented in this study shows that modern catalytically independent proteins like Rnt1p could easily be adapted for an RNP-like function. The kinetics of Rnt1p-guided cleavages indicated that the enzyme recycles to cleave several guide/target complexes (Table 2). This suggests that the artificial Rnt1p/guide complex is not as stable as known natural RNP complexes like snoRNPs for example. Therefore, if natural Rnt1p/guide complexes exist in yeast, they are probably stabilized by other protein chaperones or through RNA features that stabilizes the RNP complex[32]. However, it is not clear how established proteins would acquire the affinity for these novel RNAs. We propose that the maturation product that is normally wasted (e.g. hairpins generated as processing by-products) may be recycled into protein binding sites leading to the evolution of stable RNP complexes. Indeed, Rnt1p natural cleavage products possess the features necessary to function as guide RNAs. They contain intact Rnt1p binding sites and single stranded extensions that may function as homing devices[9], [14], [15]. To function, Rnt1p cleavage products only need protection from exonucleases and a target sequence to cleave. Indeed, vertebrate pri-miRNAs, which are essentially stem-loop structures similar to Rnt1p substrates, are processed by paralogues of RNase III, to mature into effective guides for RNA degradation and translation repression[34].

### Yeast transfection: New applications for an old model

Yeast is the most studied eukaryotic model mainly because of the powerful genetic and molecular biology tools available for both gene and genome analysis[35], [36]. Here we present a method by which small RNA molecules could be transiently introduced into yeast and the effect on RNA could be monitored independent of any effects or limitations that often come with transformation based methods. In some cases, it is very difficult to express small RNA (100 nt or less) in yeast as RNA expression from a Pol II promoter often leads to transcript polyadenylation, transport to the cytoplasm and rapid degradation. Pol III based strategies are more successful but RNA with single-stranded ends are also rapidly degraded and require the addition of a special structure that may alter the anticipated RNA activity. Direct RNA electroporation circumvents most of these problems and may be used to identify new chemistry for oligonucleotide-based gene silencing or to study the kinetics of RNA degradation *in vivo*. Chemically modified RNA like the 2′-O-methylribonucleotide form that is very popular for gene silencing in mammalian cells may now be successfully introduced using electroporation and could be tested for the first time in yeast.

### RNase IIIs tools for gene silencing

Currently, RNAi is the most successful method for design-based gene silencing. In vertebrates and many eukaryotes the introduction of short RNA duplex with sequence specific to any target genes of choice has various success rates. However, the main problem with this approach is target specificity and secondary effects triggered by the introduction of dsRNA or by the induction of the RNAi mechanism. In contrast, targeting RNA for cleavage using guide RNA uses a largely single-stranded RNA and introduces a single cleavage site that leads to RNA degradation using the normal degradation machinery (e.g. exosome). Thus, this method should reduce secondary effects associated with RNAi. In yeast, this method is restricted to nuclear RNA since Rnt1p is localized in the nucleus[37]. The nuclear specificity of this approach distinguishes it from other available approaches that appear to be active in more than one cellular compartment. Similar nuclear degradation strategies in mammalian cells may also be envisioned. It is established that Drosha, the mammalian paralogue of Rnt1p, cleaves a stem-loop structure analogous to that of Rnt1p. Thus, it is possible to imagine a similar strategy using the Drosha recognition signal to direct cleavage in independent RNA species.

## Materials and Methods

### Strains and plasmids

Yeast was grown and manipulated according to standard procedures[38], [39]. All the experiments were performed using the yeast strain YHM111 (*MATa, trp1, ura3-52, ade2-101, his3, lys2, snr20*::LYS2)[40]. The plasmids pRS314/U2 and pRS314/U2δStem were generated by subcloning *Pvu* II fragments from pRS315/U2 and pRS315/U2δStem[9] into pRS314. The U2/target RNA was expressed from pRS314/U2δStem/L2, which was generated by inserting a synthetic 36 bp dsDNA oligonucleotide (5′-CTAGAAGCTAATGTTTTGGCCTCTTCAAGATTATGG-3′) into the *Nhe*I site of pRS314/U2δStem. The strain YHM111 was transformed with pRS314/U2 or pRS314/U2δStem/L2 to yield strains YHM111-U2 and YHM111-U2L2.

### Enzymatic assays

Recombinant Rnt1p, Pac1, and *E.coli* RNase III were produced in bacteria and purified as described before[41]. Recombinant human Dicer was purchase from Stratagene (La Jolla, CA). The RNA transcripts used for the cleavage and binding assays were generated by T7 RNA polymerase using oligonucleotides as templates. Some transcripts including the EL3′11dT were chemically synthesized and purchased from Integrated DNA Technologies (Coralville, IA). The RNA transcripts derived from T7 RNA polymerase were dephosphorylated using antarctic phosphatase (New England Biolabs, Ipswich, MA) and 5′-end labeled using [γ-^32^P] ATP as described[17]. *In vitro* cleavages were performed by incubating the different RNA substrates with 20–80 nM of enzyme for 20 minutes at 30°C (or 37°C for Dicer). The reactions were carried out in 20 µl of cleavage buffer[41] for Rnt1p, Dicer and *E. coli* RNase III. For Pac1, the substrates were incubated in the presence of 80 nM of enzyme for 20 minutes at 30°C in 20 µl reaction buffer[14]. Cleavage comparison between Rnt1p and bacterial RNase H (USB, OH) in total RNA were performed using 80 nM of enzymes. All experiments were repeated three times and the average calculated. All kinetic calculations were performed using the Graph Pad Prism 4.0 program (GraphPad Software, CA).

### RNA Gel mobility Shift Assay

RNA binding experiments were performed using guide concentrations that ranged between 0.08 and 0.64 µM and 0.32 µM of unlabelled target RNA spiked with a trace of labeled target in 20 µl reaction buffer (30 mM Tris pH 7.5, 150 mM KCl, 5 mM spermidine, 10 mM MgCl_2_, 0.1 mM dithiothreitol (DTT), and 0.1 mM EDTA pH 7.5) for 5 minutes at 30°C. After incubation, 20% glycerol was added and 4 µl of each reaction was fractionated on 12% non-denaturing polyacrylamide gels. Both bound and unbound RNA fractions were quantified using Instant Imager (Packard, Meriden, CT). Each experiment was repeated twice.

### Protein Gel mobility Shift Assay

Protein binding experiments were conducted essentially as described before[12] with 3 fmol of 5′-end labeled RNA. For the *trans* reaction, 3 fmol of 5′-end labeled RNA guide was incubated with 2 pmol of cold RNA target prior to the incubation with Rnt1p. Experiments were repeated three times.

### Yeast extracts preparation

YHM111-U2 and YHM111-U2L2 strains were grown in Yeast Complete media without tryptophan (YC-trp). The growing cells were collected and the extracts prepared as previously described[12].

### Primer extension

Primer extension was performed essentially as described before[42]. Briefly, 5 µg of total RNA was incubated with 1 ng of 5′-end radiolabeled primer. The extensions in figures 3 and 4 were performed using U2/3′-end oligonucleotide (5′-TTACATATTGGTTGC-3′)[9], while those in figures 5 and 6 were performed using the U2-Br-PE oligonucleotide (5′-GGGTGCCAAAAAATGTG-3′).

### Northern Blot

The northern blots were performed essentially as described before[9]. The RNA was extracted from yeast cells and 10 µg was loaded on 1.2% agarose-formaldehyde gel and transferred to a nylon membrane (Hybond-XL, Amersham). The RNA was visualized using radiolabeled oligonucleotide probes complementary to U2 snRNA (5′-GGGTGCCAAAAAATGTG-3′), RPR1 (5′-GGGCCAATGCCAAAAGCGACATTAACCCGG-3′) and rRNA 25S (5′-ATCGACTAACCCACGTCCAACTGCTGTTGACGTGG-3′).

### RNase protection assay

A probe complementary to the 3′-end of U2L2 was derived from T7 transcription of the plasmid pRS314/U2δStem/L2 digested with *Dra*I, 97 nt bases upstream the mature U2 3′-end. The probe covers 97 bases in the mature U2 snRNA and 480 bases downstream of the mature 3′-end. A probe complementary to actin was derived from T7 transcription of the plasmid pKS/Actin digested with *Hind*III. Total yeast RNA (5 µg) was incubated at 42°C for 12 h with ∼10^5^ C.P.M. of probe in 80% formamide hybridization buffer[9]. The hybridization mix was digested with 100 U/ml RNase T1 for 1 hour at 30°C, and the protected fragments were separated on 8% denaturing acrylamide gel.

### RNA electroporation in yeast living cells

Electrocompetent cells were prepared as described before[22] with modifications. Yeast strains YHM111-U2 and YHM111-U2L2 were grown overnight in 500 ml of YC-Trp at 30°C to an OD_600_ of 0.8. The culture was chilled on ice for 30 min and centrifuged at 5000 rpm for 5 min at 4°C. The supernatant was discarded and the pellet was washed twice in 50 ml ice-cold sterile water. After the second centrifugation, the pellet was resuspended in 20 ml ice-cold 1M sorbitol and centrifuged at 5000 rpm for 5 min at 4°C. The final pellet was resuspended in 500 µl of 1M sorbitol and used directly for electroporation. For electroporation, 40 µl of yeast suspension per transformation was used with RNA guide (0.2 to 4 nmols). The pulse was performed at 1.5 kV, 25 µF, and 200 Ω with the Bio-Rad MicroPulser (Bio-Rad, Richmond, CA). Immediately after the pulse, 1 ml of ice -cold 1M sorbitol was added and transferred into a tube containing 4 ml of YC-trp media for incubation at 30°C.

## Supporting Information

Figure S1 Mapping guide-induced RNA cleavage by RNase IIIs. The substrate 3′-Branch and the RNA target (TL) in the RNA/target complexes EL9-11:TL and EL3′-11:TL were 5′-end labeled and incubated with Rnt1p (A), bacterial RNase III (B), S. pombe Pac1 (C), and human Dicer (D) in presence of Mg2+ and the cleavage products were mapped. The black and gray arrowheads indicate cleavage sites when the reactions were performed at 10 and 150 mM monovalent salt concentration respectively.
(0.23 MB TIF)

## References

[1] Tijsterman M, Plasterk RH (2004). Dicers at RISC; the mechanism of RNAi.. Cell.

[2] Xia H, Mao Q, Paulson HL, Davidson BL (2002). siRNA-mediated gene silencing in vitro and in vivo.. Nat Biotechnol.

[3] Lamontagne B, Larose S, Boulanger J, Elela SA (2001). The RNase III family: a conserved structure and expanding functions in eukaryotic dsRNA metabolism.. Curr Issues Mol Biol.

[4] Wilson HR, Yu D, Peters HK, Zhou JG, Court DL (2002). The global regulator RNase III modulates translation repression by the transcription elongation factor N.. Embo J.

[5] Ge D, Lamontagne B, Elela SA (2005). RNase III-Mediated Silencing of a Glucose-Dependent Repressor in Yeast.. Curr Biol.

[6] Larose S, Laterreur N, Ghazal G, Gagnon J, Wellinger RJ (2007). RNase III-dependent Regulation of Yeast Telomerase.. J Biol Chem.

[7] Lee A, Henras AK, Chanfreau G (2005). Multiple RNA surveillance pathways limit aberrant expression of iron uptake mRNAs and prevent iron toxicity in S. cerevisiae.. Mol Cell.

[8] Gan J, Tropea JE, Austin BP, Court DL, Waugh DS (2006). Structural insight into the mechanism of double-stranded RNA processing by ribonuclease III.. Cell.

[9] Abou Elela S, Ares M (1998). Depletion of yeast RNase III blocks correct U2 3′ end formation and results in polyadenylated but functional U2 snRNA.. Embo J.

[10] Abou Elela S, Igel H, Ares M (1996). RNase III cleaves eukaryotic preribosomal RNA at a U3 snoRNP-dependent site.. Cell.

[11] Ghazal G, Ge D, Gervais-Bird J, Gagnon J, Abou Elela S (2005). Genome-wide prediction and analysis of yeast RNase III-dependent snoRNA processing signals.. Mol Cell Biol.

[12] Lamontagne B, Hannoush RN, Damha MJ, Aboul Elela S (2004). Molecular requirements for duplex recognition and cleavage by eukaryotic RNase III: discovery of an RNA-dependent DNA cleavage activity by yeast Rnt1p.. J Mol Biol.

[13] Chanfreau G, Buckle M, Jacquier A (2000). Recognition of a conserved class of RNA tetraloops by Saccharomyces cerevisiae RNase III.. Proc Natl Acad Sci U S A.

[14] Lamontagne B, Elela SA (2004). Evaluation of the RNA determinants for bacterial and yeast RNase III binding and cleavage.. J Biol Chem.

[15] Lebars I, Lamontagne B, Yoshizawa S, Aboul-Elela S, Fourmy D (2001). Solution structure of conserved AGNN tetraloops: insights into Rnt1p RNA processing.. Embo J.

[16] Ghazal G, Elela SA (2006). Characterization of the reactivity determinants of a novel hairpin substrate of yeast RNase III.. J Mol Biol.

[17] Lamontagne B, Ghazal G, Lebars I, Yoshizawa S, Fourmy D (2003). Sequence dependence of substrate recognition and cleavage by yeast RNase III.. J Mol Biol.

[18] Chanfreau G, Elela SA, Ares M, Guthrie C (1997). Alternative 3′-end processing of U5 snRNA by RNase III.. Genes Dev.

[19] Kiss T (2002). Small nucleolar RNAs: an abundant group of noncoding RNAs with diverse cellular functions.. Cell.

[20] Hammond SM, Boettcher S, Caudy AA, Kobayashi R, Hannon GJ (2001). Argonaute2, a link between genetic and biochemical analyses of RNAi.. Science.

[21] Takei Y, Kadomatsu K, Itoh H, Sato W, Nakazawa K (2002). 5′-,3′-inverted thymidine-modified antisense oligodeoxynucleotide targeting midkine. Its design and application for cancer therapy.. J Biol Chem.

[22] Becker DM, Guarente L (1991). High-efficiency transformation of yeast by electroporation.. Methods Enzymol.

[23] Ares M (1986). U2 RNA from yeast is unexpectedly large and contains homology to vertebrate U4, U5, and U6 small nuclear RNAs.. Cell.

[24] Vickers TA, Koo S, Bennett CF, Crooke ST, Dean NM (2003). Efficient reduction of target RNAs by small interfering RNA and RNase H-dependent antisense agents. A comparative analysis.. J Biol Chem.

[25] Wintersberger U, Frank P (2001). Ribonucleases H of the budding yeast, Saccharomyces cerevisiae.. Methods Enzymol.

[26] Riedel N, Wise JA, Swerdlow H, Mak A, Guthrie C (1986). Small nuclear RNAs from Saccharomyces cerevisiae: unexpected diversity in abundance, size, and molecular complexity.. Proc Natl Acad Sci U S A.

[27] Davies J, von Ahsen U, Wank H, Schroeder R (1992). Evolution of secondary metabolite production: potential roles for antibiotics as prebiotic effectors of catalytic RNA reactions.. Ciba Found Symp.

[28] Cech TR (1989). RNA as an enzyme.. Biochem Int.

[29] Jeffares DC, Poole AM, Penny D (1998). Relics from the RNA world.. J Mol Evol.

[30] Poole AM, Jeffares DC, Penny D (1998). The path from the RNA world.. J Mol Evol.

[31] Mattick JS, Makunin IV (2005). Small regulatory RNAs in mammals.. Hum Mol Genet.

[32] Lafontaine DL, Tollervey D (1998). Birth of the snoRNPs: the evolution of the modification-guide snoRNAs.. Trends Biochem Sci.

[33] Lin SL, Ying SY (2006). Gene silencing in vitro and in vivo using intronic microRNAs.. Methods Mol Biol.

[34] Murchison EP, Hannon GJ (2004). miRNAs on the move: miRNA biogenesis and the RNAi machinery.. Curr Opin Cell Biol.

[35] Bartlett R, Nurse P (1990). Yeast as a model system for understanding the control of DNA replication in Eukaryotes.. Bioessays.

[36] Madeo F, Engelhardt S, Herker E, Lehmann N, Maldener C (2002). Apoptosis in yeast: a new model system with applications in cell biology and medicine.. Curr Genet.

[37] Catala M, Lamontagne B, Larose S, Ghazal G, Elela SA (2004). Cell cycle-dependent nuclear localization of yeast RNase III is required for efficient cell division.. Mol Biol Cell.

[38] Guthrie C, Fink GR (1991). Guide to Yeast Genetics and Molecular Biology..

[39] Rose MD, Winston F, Hieter P (1990). Methods in Yeast Genetics: A Laboratory Course Manual..

[40] Madhani HD, Guthrie C (1992). A novel base-pairing interaction between U2 and U6 snRNAs suggests a mechanism for the catalytic activation of the spliceosome.. Cell.

[41] Lamontagne B, Abou Elela S (2001). Purification and characterization of Saccharomyces cerevisiae Rnt1p nuclease.. Methods Enzymol.

[42] Ares M, Igel AH (1990). Lethal and temperature-sensitive mutations and their suppressors identify an essential structural element in U2 small nuclear RNA.. Genes Dev.

