# Supplementary figures and images for: Short RNA Guides Cleavage by Eukaryotic RNase III
Source: PLoS One. 2007 May 30;2(5):e472. doi: 10.1371/journal.pone.0000472 (PMC1868780; doi:10.1371/journal.pone.0000472)

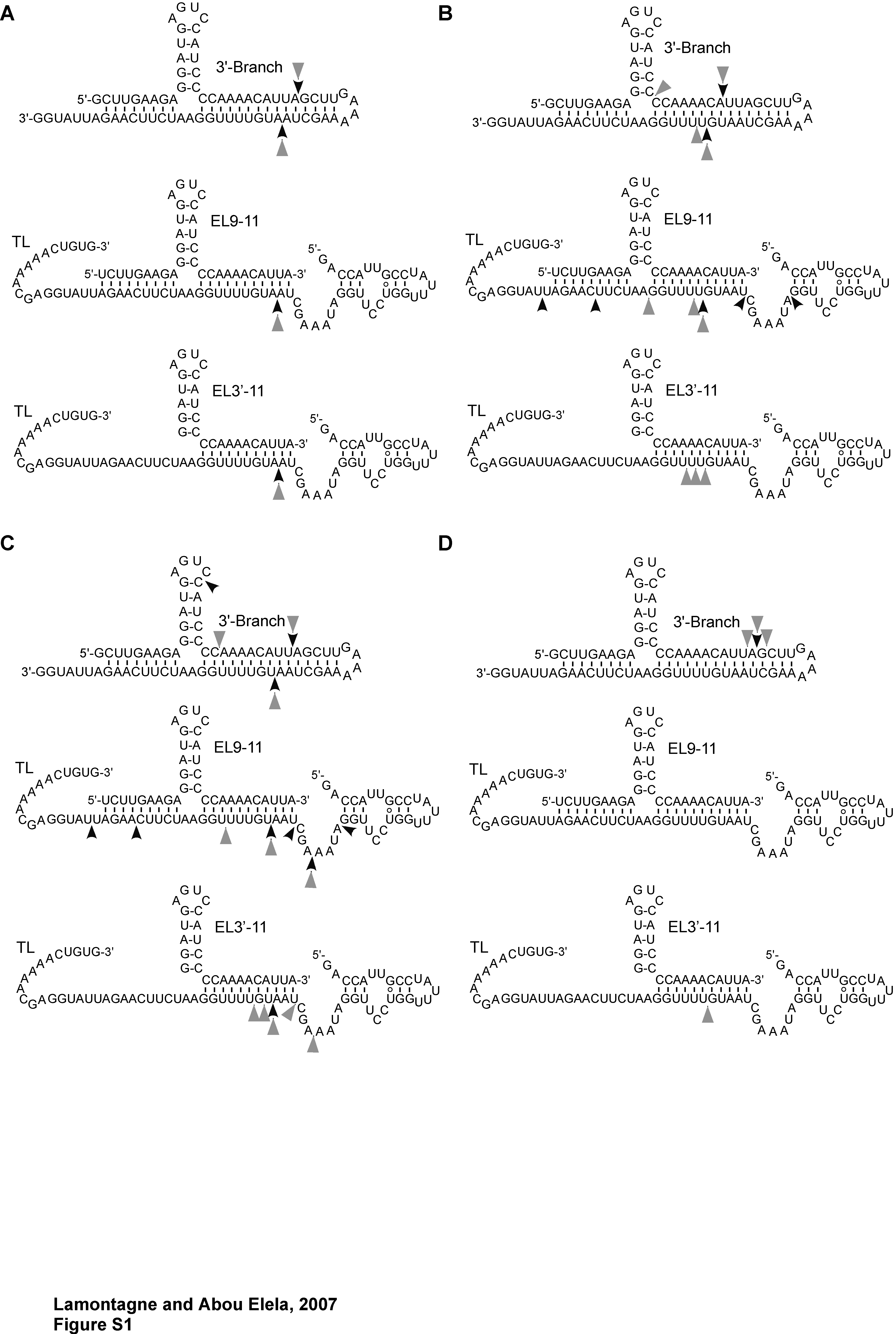

Supplement: Figure S1 — Mapping guide-induced RNA cleavage by RNase IIIs. The substrate 3′-Branch and the RNA target (TL) in the RNA/target complexes EL9-11:TL and EL3′-11:TL were 5′-end labeled and incubated with Rnt1p (A), bacterial RNase III (B), S. pombe Pac1 (C), and human Dicer (D) in presence of Mg2+ and the cleavage products were mapped. The black and gray arrowheads indicate cleavage sites when the reactions were performed at 10 and 150 mM monovalent salt concentration respectively. (0.23 MB TIF) [file pone.0000472.s001.tif]
